# Interpretable Machine Learning to Predict the Malignancy Risk of Follicular Thyroid Neoplasms in Extremely Unbalanced Data: Retrospective Cohort Study and Literature Review

**DOI:** 10.2196/66269

**Published:** 2025-02-10

**Authors:** Rui Shan, Xin Li, Jing Chen, Zheng Chen, Yuan-Jia Cheng, Bo Han, Run-Ze Hu, Jiu-Ping Huang, Gui-Lan Kong, Hui Liu, Fang Mei, Shi-Bing Song, Bang-Kai Sun, Hui Tian, Yang Wang, Wu-Cai Xiao, Xiang-Yun Yao, Jing-Ming Ye, Bo Yu, Chun-Hui Yuan, Fan Zhang, Zheng Liu

**Affiliations:** 1 Department of Maternal and Child Health, School of Public Health, Peking University, Beijing, China; 2 Department of General Surgery, Peking University Third Hospital, Beijing, China; 3 Department of Ultrasound, Peking University People's Hospital, Beijing, China; 4 Department of Thyroid and Breast Surgery, Peking University First Hospital, Beijing, China; 5 Department of Pathology, Peking University People's Hospital, Beijing, China; 6 The Key Laboratory of Experimental Teratology, Ministry of Education and Department of Pathology, School of Basic Medical Sciences, Jinan, China; 7 Department of Ultrasound, Peking University Third Hospital, Beijing, China; 8 National Institute of Health Data Science, Peking University, Beijing, China; 9 Advanced Institute of Information Technology, Peking University, Beijing, China; 10 Institute of Advanced Clinical Medicine, Peking University, Beijing, China; 11 Department of Pathology, Peking University Third Hospital, Beijing, China; 12 School of Basic Medical Sciences, Peking University Health Science Center, Beijing, China; 13 Information Management and Big Data Center, Peking University Third Hospital, Beijing, China; 14 Department of Cardiovascular Medicine, First Affiliated Hospital of Xi’an Jiaotong University, Xi'an, China

**Keywords:** follicular thyroid neoplasm, machine learning, prediction model, malignancy, unbalanced data, literature review

## Abstract

**Background:**

Diagnosing and managing follicular thyroid neoplasms (FTNs) remains a significant challenge, as the malignancy risk cannot be determined until after diagnostic surgery.

**Objective:**

We aimed to use interpretable machine learning to predict the malignancy risk of FTNs preoperatively in a real-world setting.

**Methods:**

We conducted a retrospective cohort study at the Peking University Third Hospital in Beijing, China. Patients with postoperative pathological diagnoses of follicular thyroid adenoma (FTA) or follicular thyroid carcinoma (FTC) were included, excluding those without preoperative thyroid ultrasonography. We used 22 predictors involving demographic characteristics, thyroid sonography, and hormones to train 5 machine learning models: logistic regression, least absolute shrinkage and selection operator regression, random forest, extreme gradient boosting, and support vector machine. The optimal model was selected based on discrimination, calibration, interpretability, and parsimony. To address the highly imbalanced data (FTA:FTC ratio>5:1), model discrimination was assessed using both the area under the receiver operating characteristic curve and the area under the precision-recall curve (AUPRC). To interpret the model, we used Shapley Additive Explanations values and partial dependence and individual conditional expectation plots. Additionally, a systematic review was performed to synthesize existing evidence and validate the discrimination ability of the previously developed Thyroid Imaging Reporting and Data System for Follicular Neoplasm scoring criteria to differentiate between benign and malignant FTNs using our data.

**Results:**

The cohort included 1539 patients (mean age 47.98, SD 14.15 years; female: n=1126, 73.16%) with 1672 FTN tumors (FTA: n=1414; FTC: n=258; FTA:FTC ratio=5.5). The random forest model emerged as optimal, identifying mean thyroid-stimulating hormone (TSH) score, mean tumor diameter, mean TSH, TSH instability, and TSH measurement levels as the top 5 predictors in discriminating FTA from FTC, with the area under the receiver operating characteristic curve of 0.79 (95% CI 0.77‐0.81) and AUPRC of 0.40 (95% CI 0.37-0.44). Malignancy risk increased nonlinearly with larger tumor diameters and higher TSH instability but decreased nonlinearly with higher mean TSH scores or mean TSH levels. FTCs with small sizes (mean diameter 2.88, SD 1.38 cm) were more likely to be misclassified as FTAs compared to larger ones (mean diameter 3.71, SD 1.36 cm). The systematic review of the 7 included studies revealed that (1) the FTA:FTC ratio varied from 0.6 to 4.0, lower than the natural distribution of 5.0; (2) no studies assessed prediction performance using AUPRC in unbalanced datasets; and (3) external validations of Thyroid Imaging Reporting and Data System for Follicular Neoplasm scoring criteria underperformed relative to the original study.

**Conclusions:**

Tumor size and TSH measurements were important in screening FTN malignancy risk preoperatively, but accurately predicting the risk of small-sized FTNs remains challenging. Future research should address the limitations posed by the extreme imbalance in FTA and FTC distributions in real-world data.

## Introduction

Globally, thyroid neoplasms are becoming increasingly prevalent [1]. Among them, follicular thyroid neoplasms (FTNs) represent a major type but have garnered significantly less attention compared to papillary thyroid carcinoma. A key challenge is that over 95% of FTN cases cannot be reliably distinguished as benign (follicular thyroid adenoma [FTA]) or malignant (follicular thyroid carcinoma [FTC]) until diagnostic surgery [2]. This uncertainty often leads to both over- and undertreatment of patients with FTN. On one hand, it is estimated that over 80% of patients who undergo thyroidectomy might ultimately be diagnosed as benign FTN based on postoperative pathology [3]. On the other hand, those with malignant FTN may have already developed distant metastases to the lungs, bones, or other organs by the time they receive surgical treatment.

Several guidelines advocate for enhanced screening, accurate diagnosis, and appropriate treatment for patients with FTN [4,5]. One crucial solution is to develop prediction models to aid clinical decision-making for these patients. To date, machine learning has been proven effective in constructing predictive models for various cancers such as oral, gastrointestinal, and breast cancers [6-8]. Our literature review also indicated that machine learning technology excels at capturing complex, nonlinear relationships and high-dimensional intercorrelations among predictors [9-12].

However, our literature review revealed several limitations among most of the existing studies, mainly including (1) small sample sizes ranging from 18 to 888 participants [13-19], (2) the ratio of FTA to FTC deviating from the real population distributions, (3) reliance on simple linear models unable to capture the complex nonlinearity or interactions underlying predictor-outcome relationships [14,18], (4) using inappropriate metrics to evaluate model performance for the unbalanced data [13-19], (5) lack of assessing the extent to which a predictor influences the model’s prediction (ie, model interpretability) [13,16,19], (6) not evaluating whether the predicted probabilities were consistent with actual outcomes (ie, model calibration) in the development and validation of clinical prediction models [20], and (7) predictors are predominantly confined to sonographic features with limited consideration of other factors such as the presence of Hashimoto thyroiditis (an autoimmune disease that may increase the risk for differentiated thyroid cancer [21]).

To address these limitations, our study has united a multidisciplinary treatment team for thyroid neoplasms and accumulated a cohort of over 1500 patients with FTN over the past decade [22]. This provided us a unique opportunity to develop and validate clinical prediction models to bridge the current research gaps in the field of FTN. Specifically, we aimed to individualize the clinical decision-making for patients with FTN by using interpretable machine learning to not only predict the malignancy risk of FTN but also identify the important predictors that might contribute to the prediction.

## Methods

### Study Design

**Figure 1.**
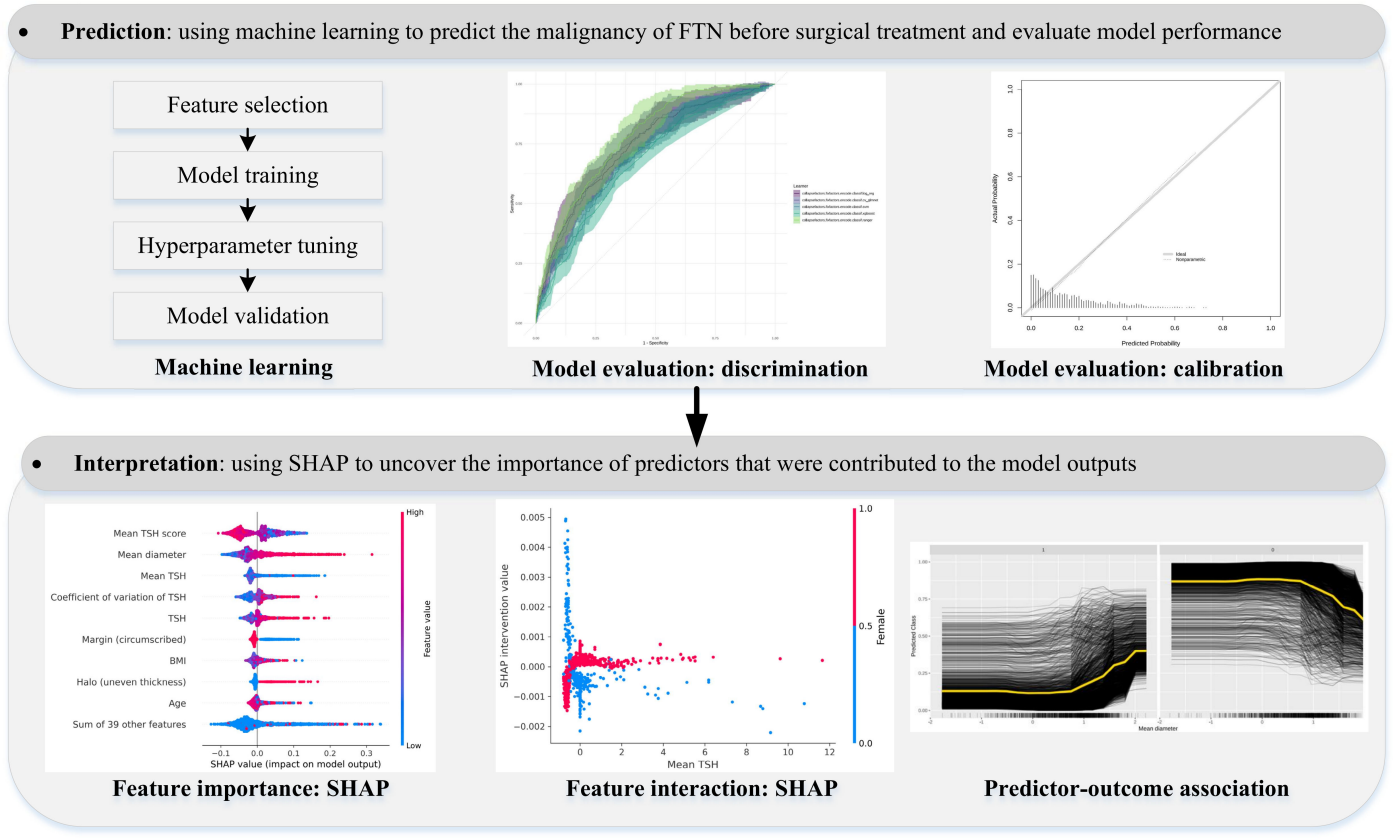
Study framework of the machine learning–based modeling to facilitate the clinical decision-making for patients of FTN. FTN: follicular thyroid neoplasm; SHAP: Shapley Additive Explanations; TSH: thyroid-stimulating hormone.

This retrospective cohort study followed the suggestions of the TRIPOD (Transparent Reporting of a Multivariable Prediction Model for Individual Prognosis or Diagnosis) statement [23]. Figure 1 shows the framework of our study.

### Study Population

Our multidisciplinary research team included experts in the fields of epidemiology, surgery, pathology, ultrasound, and endocrinology. We conducted a retrospective cohort study at Peking University Third Hospital in Beijing, China, from January 2012 to September 2023. Eligible patients were those who underwent surgery and were pathologically diagnosed with FTA or FTC following the procedure. Patients were excluded if they did not undergo ultrasound examinations prior to surgery or if they had nodules classified as follicular tumors of uncertain malignant potential (UMPs). This exclusion was based on two considerations: (1) accurate diagnosis of FTNs required both an experienced pathologist and a complete biopsy sample. The key to distinguishing between benign and malignant FTNs was determining whether the tumor invaded the capsule. Tumors that invaded the capsule or blood vessels were classified as FTC, while those that did not were considered FTA. If the pathologist struggled to assess capsule invasion due to inexperience, or if the sample was inadequately collected during surgery, leading to capsule damage, the tumor might not have been accurately classified as either FTA or FTC. In such cases, it could have been labeled as a UMP. (2) Through a literature review, we found that all previous research had excluded UMPs [13-19]. Therefore, our study also excluded UMPs, enhancing comparability with prior research. We paid close attention to the accuracy of the pathological diagnosis of FTN due to its high professional requirements, which include not only complete sampling but also a thorough examination of all areas of the tumor margin. To ensure this, we invited pathologists with expertise in thyroid tumors to double-check all the postoperational pathological diagnoses in the study population, based on the most recent 2022, 5th edition WHO Classification of Thyroid Neoplasms [24].

It is important to note that our study population reflected the natural distribution of FTNs (ie, the ratio of FTA and FTC), resulting in imbalanced data, with 84.57% (n=1414) of cases was FTA. Specifically, we did not restrict the ratio of FTA to FTC to 1:1 or any other fixed ratio in the main analyses. This approach allowed the results from the developed prediction model to be more readily applicable to external populations with a similar natural distribution.

### Data Sources and Processing

The data for this study were sourced from the electronic health records, extracted by professional information management personnel from the hospital’s electronic information system. For critical data sources like thyroid pathology and neck ultrasound reports, a tailored data extraction form was designed using EpiData (EpiData Association), aligning with the study’s research questions. The form was iteratively refined through discussions among researchers, surgeons, ultrasound specialists, and pathologists, followed by trial entries and revisions until finalized. Trained clinical doctors and medical students performed manual data entry, with researchers conducting 2 rounds of random checks to ensure accuracy and consistency. Senior doctors performed a final review to verify data quality. Missing values were imputed using the mean for continuous variables and the mode for categorical variables.

### Predictors

We selected the predictors to develop the machine learning–based model based on our systematic review [17,18], domain knowledge [25-27], and data available. The predictors included sonographic features, patients’ age, sex, BMI, whether or not diagnosed as Hashimoto thyroiditis (an autoimmune disease that destroys thyroid cells by cell and antibody-mediated immune processes [28]), and measurements of thyroid hormones. Specifically, the sonographic features included mean diameter, composition (solid, predominantly solid, predominantly cystic, or cystic), echogenicity (hyperechoic, isoechoic, hypoechoic, or anechoic), taller-than-wide (the length in the vertical direction is greater than the width in the horizontal direction: absent or present), margin (circumscribed, ill-defined, irregular, or lobulated), calcifications (microcalcifications, macrocalcifications, peripheral calcifications, punctate echogenic foci of undetermined significance, microcalcifications with comet-tail artifacts, or no echogenic foci), halo (absent halo, even thickness halo, uneven thickness halo, or present halo without evenness of thickness reported), internal blood flow (absent or present), vascularity (mainly central vascularity, mainly peripheral vascularity, mixed vascularity, or avascularity), trabecular formation (typically appears as elongated, band-like, or fibrous echogenicity, arranged in a reticular or cord-like pattern: absent or present), and nodule-in-nodule appearance (a smaller nodule or an area with different echogenic characteristics is present within a larger thyroid nodule: absent or present); the measurements at the latest examination of thyroid hormones included thyroid-stimulating hormone (TSH), free triiodothyronine, and free thyroxine; additionally, TSH-related features derived from all examinations included mean TSH score (interval-adjusted detailed TSH score) [29], time-adjusted root mean square of successive differences of TSH [30], mean TSH (mean value of preoperative TSH), and coefficient of variation of TSH (the ratio of SD of preoperative TSH to mean value of preoperative TSH), and detailed definitions were introduced in previous publication [31]. All selected predictors were carefully checked by both clinicians and researchers to ensure the accuracy and reliability of the study results.

### Development and Validation of Machine Learning–Based Models

We established the machine learning–based model as shown in Figure 1. We selected features, trained models, tuned hyperparameters, and validated models, as briefly described below. We used the mlr3 [32] ecosystem in R (version 4.3.3; R Foundation for Statistical Computing), scikit-learn [33], and Shapley Additive Explanations (SHAP) [34] in Python (version 3.11.1; Python Software Foundation) to conduct machine learning.

Feature selection, which aims to reduce the number of features, offers several benefits including minimizing overfitting, enhancing model robustness, and accelerating predictions. Notably, it is particularly advantageous for datasets with a high feature-to-sample ratio, where the number of features exceeds the limited size of data points. To identify a core set of predictors that could effectively predict the outcome without redundancy, we used a novel information-gain approach for feature selection [35]. To finalize the optimal model, we also compared model performance between that with full predictors and that with selected predictors.

We trained 5 classification models including logistic regression, least absolute shrinkage and selection operator (LASSO) regression, random forest, extreme gradient boosting, and support vector machine. We comprehensively considered and weighed (trade-off) the performance, calibration, parsimony, and interpretability of models and selected the most appropriate one as our prediction model.

The random search and cross-validation were combined to select model hyperparameters when training the machine learning model. We performed a random search over more than 45,000 hyperparameter combinations to select the best hyperparameter combination and trained the final classifiers. Additionally, to address the issue of imbalance, both oversampling (increasing the amount of minority class samples with producing new samples or repeating some samples) and undersampling (decreasing the amount of majority class samples) techniques were applied [36].

### Evaluation of Model Performance

We evaluated the performance of the developed model in terms of discrimination (the ability of the model to distinguish between those with and without the outcome) and calibration (the consistency or agreement between the observed outcomes and predicted risks from the model). For discrimination, we first showed the confusion matrix including the numbers and percentages of true positive, true negative, false positive, and false negative. We then calculated both the threshold-free and threshold-sensitive metrics. Threshold-free metrics included the area under the receiver operating characteristic curve (AUROC) and the area under the precision-recall curve (AUPRC). While AUROC was a common performance metric for discrimination, AUPRC was considered more useful and informative for handling the unbalanced data in this study [37]. Threshold-sensitive metrics included sensitivity, precision, specificity, and accuracy. For calibration, we first plotted predicted risks (x-axis) against observed outcomes (y-axis) using a smoothed flexible calibration curve based on individual data. We also quantitatively assessed calibration using the calibration slope and calibration-in-the-large.

### Interpretation of Model Prediction Results

First, we evaluated the feature importance (ie, the extent of the model depended on the feature) and the feature interaction by using the SHAP summary plot and SHAP interaction value dependence plot (see details in Multimedia Appendix 1) [34]. Second, we figured the partial dependence plots to visualize the direction of predictor-outcome associations, illustrate whether the risk of the outcome increased with a rise or decline in the predictor values, and assess whether this relationship is linear. Third, we plotted individual conditional expectations curves to explore potential modifiers that could influence predictor-outcome associations. Finally, we separated FTC into 2 groups based on whether they were correctly predicted and compared their characteristics. The significance of differences between the groups was tested using the Mann-Whitney *U* test, as the data did not follow to a normal distribution.

### A Systematic Review of the Previous Studies

We conducted a systematic review of previous studies addressing similar topics. According to the PRISMA (Preferred Reporting Items for Systematic Reviews and Meta-Analyses) guidelines [38] (see details in Checklist 1), we searched PubMed, Web of Science, Embase, and IEEE Xplore using the terms “follicular thyroid cancer” and “predict” for papers published up to October 1, 2023 (see details in Multimedia Appendix 2). Eligible studies included those that established prediction models to distinguish FTC from FTA before operation with various preoperative predictors. We included studies with either deep learning models, machine learning models, traditional statistical models, or other relevant methodologies. Studies were excluded if fewer than 50% of patients had FTN or if the papers were not written in English.

We evaluated the Thyroid Imaging Reporting and Data System for Follicular Neoplasm (F-TIRADS) scoring criteria developed by Li et al [18] to differentiate between benign and malignant cases in our dataset. These criteria are based on 6 key features: mean diameter, composition, echogenicity, margin, calcifications, and trabecular formation. Each specific characteristic of these features is assigned a corresponding point value, and the total points across the 6 features indicate the risk level of FTC. For instance, a total score of 12 points or higher suggests an FTC risk exceeding 90% (refer to Figure S1 in Multimedia Appendix 1).

### Ethical Considerations

This study was classified as human participant research and was reviewed and approved by the medical research ethics committee of Peking University Third Hospital (IRB00006761-M2023168). As a retrospective analysis, the study was granted a waiver for additional informed consent. During the data extraction process, strict confidentiality measures were implemented to ensure patient privacy and data security. All extracted data were anonymized, with any information that could directly identify patients being removed.

## Results

### Characteristics of the Study Population

Altogether, we included 1539 patients, 1409 of whom had solitary tumors, and 130 had more than 1 tumor. Thus, a total of 1672 tumors were included and divided into 2 pathological types: FTA (n=1414) and FTC (n=258). The characteristics of the included tumors are listed in Table 1, and the characteristics of the study population are listed in Table 2. The age of the included population was 47.98 (SD 14.15) years (n=1530; missing value=9), the mean BMI was 24.18 (SD 3.66) kg/m^2^ (n=1475; missing value=64), and the female population made up 73.16% (n=1126; male: n=342; missing value=71) of all patients.

**Table 1.** Characteristics of the tumors.

Characteristics		FTA^a^	FTC^b^
Number of tumors, n (%)		1414 (84.57)	258 (15.43)
**Composition, n (%)**			
	Solid	650 (45.97)	140 (54.26)
	Predominantly solid	445 (31.47)	75 (29.07)
	Predominantly cystic	161 (11.39)	16 (6.20)
	Cystic	15 (1.06)	1 (0.39)
	N/A^c^	143 (10.11)	26 (10.08)
**Echogenicity, n (%)**			
	Anechoic	7 (0.50)	0 (0)
	Hyperechoic	31 (2.19)	7 (2.71)
	Isoechoic	660 (46.68)	105 (40.70)
	Hypoechoic	547 (38.68)	130 (50.39)
	N/A	169 (11.95)	16 (6.20)
**Margin, n (%)**			
	Circumscribed	1073 (75.88)	166 (64.34)
	Ill-defined	38 (2.69)	6 (2.33)
	Irregular	116 (8.20)	39 (15.12)
	Lobulated	69 (4.88)	35 (13.57)
	N/A	118 (8.35)	12 (4.65)
**Halo, n (%)**			
	Uneven thickness halo	149 (10.54)	55 (21.32)
	Even thickness halo	444 (31.40)	61 (23.64)
	Absent halo	602 (42.57)	108 (41.86)
	Present halo without evenness of thickness reported	83 (5.87)	11 (4.26)
	N/A	136 (9.62)	23 (8.91)
**Taller-than-wide, n (%)**			
	Absent	1174 (83.03)	214 (82.95)
	Present	73 (5.16)	18 (6.98)
	N/A	167 (11.81)	26 (10.08)
**Calcifications, n (%)**			
	No echogenic foci	1134 (80.20)	179 (69.38)
	Microcalcifications	106 (7.50)	24 (9.30)
	Macrocalcifications	117 (8.27)	40 (15.50)
	Peripheral calcifications	15 (1.06)	10 (3.88)
	Microcalcifications with comet-tail artifacts	22 (1.56)	5 (1.94)
	Punctate echogenic foci of undetermined significance	20 (1.41)	0 (0)
	N/A	0 (0)	0 (0)
**Internal blood flow, n (%)**			
	Absent	163 (11.53)	21 (8.14)
	Present	1183 (83.66)	227 (87.98)
	N/A	68 (4.81)	10 (3.88)
**Vascularity, n (%)**			
	Mainly central vascularity	69 (4.88)	17 (6.59)
	Mainly peripheral vascularity	420 (29.70)	64 (24.81)
	Mixed vascularity	578 (40.88)	143 (55.43)
	Avascularity	4 (0.28)	0 (0)
	N/A	343 (24.26)	34 (13.18)
**Trabecular formation, n (%)**			
	Absent	1224 (86.56)	227 (87.98)
	Present	30 (2.12)	15 (5.81)
	N/A	160 (11.32)	16 (6.20)
**Nodule-in-nodule appearance, n (%)**			
	Absent	1231 (87.06)	227 (87.98)
	Present	23 (1.63)	15 (5.81)
	N/A	160 (11.32)	16 (6.20)
**Mean diameter**			
	Mean (SD) (cm)	2.30 (1.17)	2.94 (1.39)
	N/A, n (%)	0 (0)	0 (0)

a FTA: follicular thyroid adenoma.

b FTC: follicular thyroid carcinoma.

c N/A: not available data.

**Table 2.** Characteristics of the study population.

Characteristics		FTA^a^	FTC^b^
**Hashimoto thyroiditis, n (%)**			
	Absent	854 (66.30)	129 (51.39)
	Present	357 (27.72)	83 (33.07)
	N/A^c^	77 (5.98)	39 (15.54)
**Sex, n (%)**			
	Male	286 (22.20)	56 (22.31)
	Female	943 (73.21)	183 (72.91)
	N/A	59 (4.58)	12 (4.78)
**Age (years)**			
	Mean (SD)	47.89 (14.05)	48.47 (14.68)
	N/A, n (%)	1 (0.08)	8 (3.19)
**BMI (kg/m** ^**2**^ **)**			
	Mean (SD)	24.08 (3.64)	24.67 (3.70)
	N/A, n (%)	59 (4.58)	5 (1.99)
**Thyroid-stimulating hormone (μIU/mL)**			
	Mean (SD)	1.76 (1.83)	1.99 (1.55)
	N/A, n (%)	325 (25.23)	90 (35.86)
**Free triiodothyronine (pg/mL)**			
	Mean (SD)	3.27 (0.66)	3.32 (0.67)
	N/A, n (%)	326 (25.31)	85 (33.86)
**Free thyroxine (ng/dL)**			
	Mean (SD)	1.27 (0.20)	1.26 (0.27)
	N/A, n (%)	325 (25.23)	85 (33.86)

a FTA: follicular thyroid adenoma.

b FTC: follicular thyroid carcinoma.

c N/A: not available data.

### Model Performance in Discrimination and Calibration

We compared performance among 5 models (logistic regression, LASSO regression, random forest, extreme gradient boosting, and support vector machine) using the AUROC and AUPRC. As shown in MultimediaAppendices 3  4, the random forest model performed better in both AUROC and AUPRC than the other 4 models. With comprehensive consideration of the discrimination, calibration, parsimony, and interpretability of models, we selected the random forest model as the optimal.

We developed a random forest model with a total of 22 features: age, sex, BMI, Hashimoto thyroiditis, thyroid hormones (TSH, free triiodothyronine, and free thyroxine), ultrasonic predictors (mean diameter, composition, echogenicity, taller-than-wide, margin, calcifications, halo, internal blood flow, vascularity, trabecular formation, and nodule-in-nodule appearance), and TSH-related variables (mean TSH score, time-adjusted root mean square of successive differences of TSH, mean TSH, and coefficient of variation of TSH). After 5-fold cross-validation, the AUROC of the prediction model was 0.79 (95% CI 0.77-0.81) and the AUPRC was 0.40 (95% CI 0.37-0.44). When the threshold is gradually lowered from 50%, 40%, 30%, 20%, and finally to 10%, the accuracy, specificity, and precision decreased step by step while the sensitivity increased progressively (Multimedia Appendix 5). The calibration slope and calibration-in-the-large were 1.16 and 0.13, respectively (Multimedia Appendix 6).

In addition, we implemented both oversampling and undersampling techniques to handle the imbalance in our models. However, following oversampling, the AUROC and AUPRC were 0.76 and 0.37, respectively, while after undersampling, the AUROC and AUPRC were 0.77 and 0.39, respectively. Notably, the model performed better before applying these sampling methods, with an AUROC of 0.79 and an AUPRC of 0.40.

### Model Performance in Interpretation

**Figure 2.**
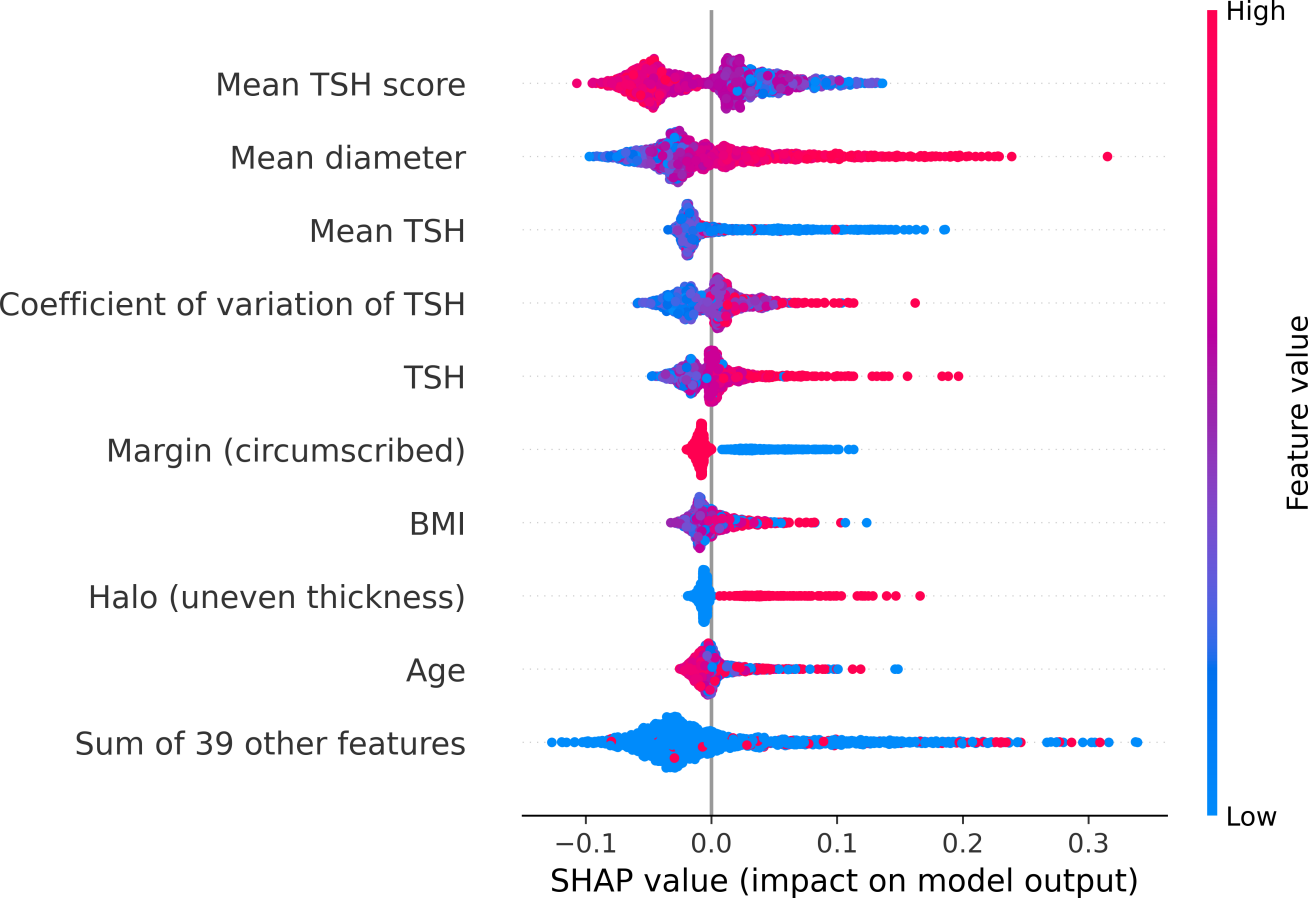
SHAP summary plot. SHAP: Shapley Additive Explanations; TSH: thyroid-stimulating hormone.

The top 5 predictors were the mean TSH score, mean tumor diameter, mean TSH, coefficient of variation of TSH, and TSH level (Figure 2). The 5 top predictors did not show explicit interactions with the sex or other predictors (Multimedia Appendix 7). Due to the strong correlation between TSH level and mean TSH score (correlation coefficient>0.6), we only plotted the partial dependence and individual conditional expectation plots for the top 4 features, excluding the TSH level. The associations between those 4 top continuous features and prediction probability were nonlinear (Multimedia Appendix 8). In general, the risk of malignancy tended to rise as the mean tumor diameter or the coefficient of variation of TSH increased, and the risk of malignancy tended to decrease as the mean TSH score or the mean TSH increased.

Moreover, we compared the characteristics of FTC groups with incorrect and correct predictions. FTC predicted as FTA by the model was classified into the incorrect-predicted group, while FTC predicted as malignant correctly by the model was then classified into the correct group. The mean diameter of the tumor was smaller in the incorrect-predicted group compared to the correct-predicted group (incorrect vs correct mean 2.88, SD cm 1.38 vs mean 3.71, SD 1.36 cm; mean diameter *W*=1474.5; *P*=.02).

### A Systematic Review of the Previous Studies

**Figure 3.**
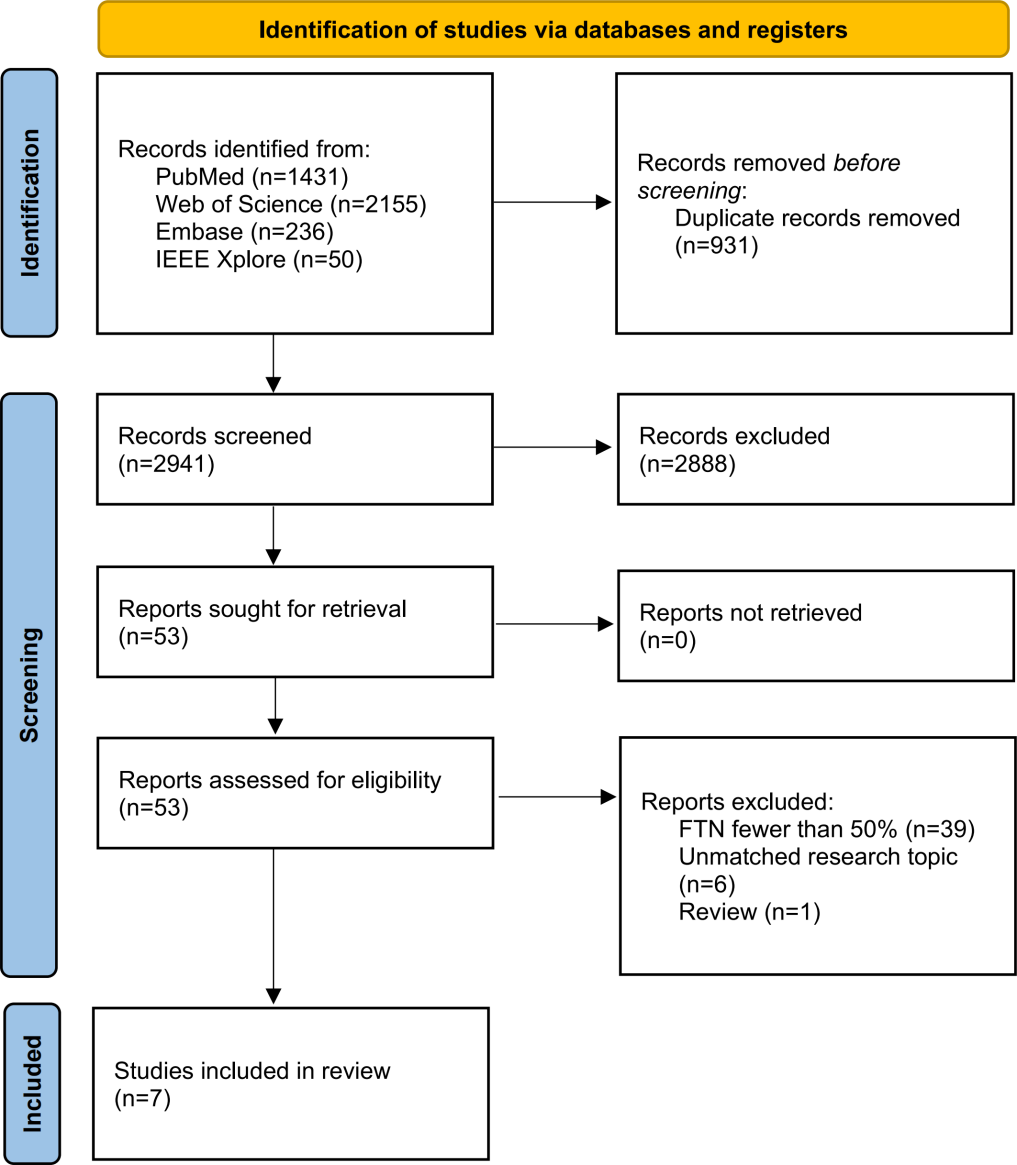
PRISMA (Preferred Reporting Items for Systematic Reviews and Meta-Analyses) study flow diagram. FTN: follicular thyroid neoplasm.

After screening citations, we eventually included 7 studies in this systematic review (refer to Figure 3). The characteristics of the included studies are presented in Table 3. The sample sizes of the studies ranged from 18 to 888 patients [13-19]. The ratio of FTA to FTC in previous studies varied from 0.64 to 4.00 [13-19], which was much smaller than the ratio observed in our study (5.50) and in the real population, where the ratio of FTA to FTC can be as high as 5:1 [3]. In total, 3 studies even set the ratio close to 1 to address the imbalance [15,17,19]. As for the model selection, 4 studies developed deep learning models [13,16,17,19], 1 study used a random forest model [15], and the other 2 studies only established linear regression models [14,18], without concerning nonlinear associations or complicated interactions. Previous studies did not use gene mutations and other biomarkers as predictive variables. Except for 1 study from South Korea, which reported an AUROC of just 0.612 [13], the AUROC of the models in the other studies ranged from 0.75 to 0.96. However, none of them used AUPRC as a metric to assess discrimination. As for the interpretation of the models, Lin et al [15] assessed the feature importance, Tang et al [14] drew a nomogram, Li et al [18] developed F-TIRADS scoring criteria, and Yang et al [17] drew a heat map to visualize the importance of pixel regions, but the other 3 studies did not further explore interpretability, including feature importance, or the linear and nonlinear associations and interactions between features and targets.

**Table 3.** Characteristics of the included studies.

Study	Country	Pathological ratio (FTA^a^/FTC^b^~ratio)	Features	Model	External test set	Sample size (n, patients)	Discrimination	Interpretation
Seo et al (2017) [16]	Korea	250/83~3.01	Ultrasound image	CNN^c^	No	Training set: 78; validation set: 229	Sensitivity: 71.05%; specificity: 93.19%; precision: 89.52%; AUROC^d^: 0.8088	No
Shin et al (2020) [13]	Korea	252/96~2.63	Ultrasound image	ANN^e^ and SVM^f^	No	Training set: 340; validation set: leave-one-out cross-validation	ANN: precision: 74.1%; sensitivity: 32.3%; specificity: 90.1%; AUROC: 0.612; SVM: precision: 69%; sensitivity: 41.7%; specificity: 79.4%; AUROC: 0.605	No
Yang et al (2020)^g^ [19]	China	Number of images: training set: 340/324~1.05; validation set: 85/81~1.05; additional test set: 154/146~1.05	Ultrasound image	CNN	No	Training set: 664 images; validation set: 166 images; test set: 300 images	Sensitivity: 95.89%; specificity: 96.10%; precision: 96%; AUROC:0.96	No
Tang et al (2021) [14]	China	112/28~4.00	Computed tomography features and clinical features and hormone level	LASSO^h^ regression	No	Training set: 140; validation set: 60	Sensitivity: 92.9%; specificity: 77.7%; precision: 80%; AUROC: 0.913 (95% CI 0.850‐0.975)	Nomogram
Li et al (2023) [18]	China	Training set: 515/188~2.74; validation set: 122/33~3.70	Ultrasound features	LASSO regression and logistic regression	No	Pretraining set: 30; training set: 703; validation set: 155	LASSO regression: sensitivity: 66%; specificity: 72%; precision: 71%; AUROC: 0.76 (95% CI 0.72‐0.79); Logistic regression: sensitivity: 64%; specificity: 75%; precision: 72%; AUROC: 0.75 (95% CI 0.71‐0.79)	F-TIRADS^i^ scoring criteria
Yang et al (2023) [17]	China	Training set: 705/687~1.02; validation set: 177/172~1.03; external test set: 150/159~0.94	Ultrasound image	CNN	Yes	Training set: 352; validation set: 80; external test set: 71	Sensitivity: 66.7%; specificity: 79.6%; precision: 73%; AUROC: 0.81 (95% CI, 0.76‐0.86)	Heat map
Lin et al (2024) [15]	United States	7/11~0.64	Ultrasound image features and clinical features	Random forest	No	Training set: 18; validation set: leave-one-out cross-validation	Sensitivity: 100%; specificity: 43%; AUROC: 0.792	Feature importance

a FTA: follicular thyroid adenoma.

b FTC: follicular thyroid carcinoma.

c CNN: convolutional neural network.

d AUROC: area under the receiver operating characteristic curve.

e ANN: artificial neural network.

f SVM: support vector machine.

g Yang et al (2020) [19] did not report the numbers of patients or nodules.

h LASSO: least absolute shrinkage and selection operator.

i F-TIRADS: Thyroid Imaging Reporting and Data System for Follicular Neoplasm.

Additionally, we tested the F-TIRADS scoring criteria developed by Li et al [18] with our dataset. The criteria specify 6 key features for scoring. After filtering our data to include only cases with complete information for these 6 features, we selected 1025 tumors from 993 patients as an external test set. When applying the F-TIRADS scoring criteria, the predictive performance was suboptimal. With a threshold for FTC risk set at >90%, the model achieved an accuracy of 0.82, sensitivity of 0.04, specificity of 0.99, and precision of 0.53. When using a >50% FTC risk threshold, the sensitivity increased to 0.27, while accuracy, specificity, and precision decreased to 0.79, 0.91, and 0.39, respectively. For threshold-independent metrics, the AUROC and AUPRC for the F-TIRADS scoring criteria were 0.47 and 0.59, respectively, in our external test set.

## Discussion

### Principal Findings

This study systematically established the interpretable machine learning–based model to address the challenge of clinical decision-making for the FTN before surgery. We developed a model using 22 readily available predictors with a preferable AUROC (0.79, 95% CI 0.77‐0.81). Additionally, the model demonstrated excellent interpretability, identifying the mean TSH score, mean tumor diameter, mean TSH, coefficient of variation of TSH, and TSH level as the most important predictors. After comparing groups of incorrect-predicted and correct-predicted FTC, we found that smaller FTCs were more likely to be misclassified as FTA.

### Comparison to Prior Work

It is crucial to evaluate the performance of clinical prediction models comprehensively, that is, the models should be well performed in discrimination and calibration. Concerning the discrimination, our developed model was comparable to that of previous studies aimed at predicting the malignancy risk of FTN before surgical treatment. For example, according to Li et al [18], the AUROC reached 0.76 in the LASSO regression model consisting of ultrasound features (the ratio of FTA to FTC: training set: 2.74 and validation set: 3.70); also, in the LASSO regression model, the AUROC reached 0.913 in discriminating FTA from FTC on selected clinical parameters, computed tomography signs, and radiomic features referring to Tang et al [14] (the ratio of FTA to FTC: 4.00). However, neither study used AUPRC as the evaluation metric. We also acknowledge that the model was derived from extremely imbalanced data (the ratio of FTA to FTC: 5.50), and in this context, the AUPRC metric for assessing model performance is more informative and intuitive than the AUROC [37]. For example, the prediction model might perform relatively well when measured by AUROC but may perform unsatisfactorily when measured by AUPRC, in the scenario of imbalanced data. Furthermore, to address the imbalance in our models, we applied oversampling and undersampling techniques, but both failed to improve performance. Our findings were in line with the previous research, which found that oversampling and undersampling generally did not enhance prediction models in large observational health datasets [39]. The possible reasons might be as follows: (1) oversampling and undersampling would modify the outcome proportions in the training data, leading to miscalibration, such as overestimated risks [39]; (2) the synthetic data generated by oversampling may not accurately represent the original distribution of minority class, potentially affecting classification performance [40]; and (3) undersampling reduced the number of majority class samples, limiting the model’s ability to fully use the features of the majority class during training [41].

As one previous systematic review indicated, calibration was commonly overlooked during the development and validation of clinical prediction models [20]. However, calibration metrics are also important to assess the size of the gap between the predicted risk probability and the true risk probability. For instance, grouping can be manipulated to obscure the evaluation of miscalibration in a particular range without a calibration curve and its numerical quantification [42]. In general, our model had relatively good calibration, as the calibration slope was close to 1 and the calibration-in-the-large close to 0.

Based on the results of our systematic review, most of the previous studies show relatively satisfying AUROC, but none of them reported AUPRC. Although Li et al [18] reported a handy score-risking tool for clinicians to assess the malignancy risk of FTN at the diagnosis stage, this tool seemed to not perform ideally in the practice of our data (AUROC 0.47; AUPRC 0.59; sensitivity 0.04 [threshold 90%] and 0.27 [threshold 50%]).

### Limitations and Strengths

We should interpret the study findings cautiously. As with other single-center studies, the results from this study were limited in generalizability to patients and clinical settings with distinct characteristics. However, the pathological diagnosis of FTC was highly heterogeneous across different clinical settings due to its challenge in sufficient sampling and accurate diagnosis. Therefore, we advocated for the standardization of FTC diagnosis before the conduction of a multicenter study soon. Additionally, the prediction performance of models, comparable to the previous work with similar predictors, had room to further improve. The clinical utility of the screening stage was also less than ideal. Building on the experiences and lessons learned from this study, we are conducting a prospective cohort study to further optimize the model performance through collecting other costly multidimensional predictors including genomics, ultrasound images, and videos. Besides, our study was retrospective in nature, which may introduce selection bias. Furthermore, we excluded patients with nodules of UMPs, potentially limiting the model’s accuracy in identifying borderline follicular tumors. Moreover, our models did not incorporate other potential predictors, such as genetic markers (eg, BRAF, TRET, and RAS mutations), computed tomography or magnetic resonance imaging characteristics, or family history, due to constraints in data availability.

Our study had several strengths. Our models were advantageous in the large sample size for the present topic, the clinically easy-accessible and clinician-validated predictors, and the comprehensive evaluation with the metrics appropriate for the nature of the data (imbalanced data) [43]. Furthermore, the disease distribution of FTA and FTC in the study population was fully consistent with that of patients with FTN in real-world settings, that is, we did not deliberately over- or undersample patients with any type of disease in the model development, as commonly seen in previous studies [17,18]. As such, findings from our study had theoretically better fidelity and generalizability in real-world settings. In addition, we conducted a systematic review to synthesize findings from previous studies, comprehensively integrate the evidence, and identify research gaps.

### Future Directions

Our study paved the way for future research in terms of predictors, models, and targets. Concerning predictors and models, further studies might consider taking advantage of the rapidly developing deep learning models and fully using high-dimension predictors such as ultrasound images and genomics. In terms of targets, it is important to standardize the pathological diagnosis of FTC across multiple centers before conducting a future multicenter study.

Our study is also important for future clinical practice. First, findings from the interpretation of our models indicate that clinicians should comprehensively consider patients’ variables such as thyroid hormones in addition to the ultrasound results. Second, in a natural distribution population with severely unbalanced data (FTA is far more than FTC), preoperative prediction of FTA and FTC by thyroid hormone and ultrasound features alone may face challenges, especially for relatively small-sized FTCs, which are easy to miss detection.

### Conclusions

In clinical practice, it remained challenging to sensitively screen, precisely diagnose, and appropriately treat patients with FTN. Interpretation of our developed machine learning–based model suggests that clinicians should also pay attention to patients’ variables such as TSH along with tumor size. However, it may be hard to correctly predict FTNs preoperatively with thyroid hormone and ultrasound features alone, especially for FTCs with small sizes. The findings of our study bridged the gaps of previous work and paved the way for connecting machine learning to interpretation in the field of FTN research. We call for subsequent studies to further examine the generalizability to other contexts.

## Supplementary material

10.2196/66269 Multimedia Appendix 1 Supplementary information for methods, definitions, and scoring criteria.

10.2196/66269 Multimedia Appendix 2 Search strategy and eligibility criteria.

10.2196/66269 Multimedia Appendix 3 Comparison between 5 different models.

10.2196/66269 Multimedia Appendix 4 Performance of 5 different models.

10.2196/66269 Multimedia Appendix 5 Area under the curve and confusion matrix (true positive, true negative, false positive, and false negative).

10.2196/66269 Multimedia Appendix 6 Calibration plot.

10.2196/66269 Multimedia Appendix 7 Shapley Additive Explanations interaction dependence plot.

10.2196/66269 Multimedia Appendix 8 Partial dependence plot and individual conditional expectation plot.

10.2196/66269 Checklist 1 PRISMA (Preferred Reporting Items for Systematic Reviews and Meta-Analyses) checklist.

## Abbreviations:

AUPRC: area under the precision-recall curve
AUROC: area under the receiver operating characteristic curve
F-TIRADS: Thyroid Imaging Reporting and Data System for Follicular Neoplasm
FTA: follicular thyroid adenoma
FTC: follicular thyroid carcinoma
FTN: follicular thyroid neoplasm
LASSO: least absolute shrinkage and selection operator
PRISMA: Preferred Reporting Items for Systematic Reviews and Meta-Analyses
SHAP: Shapley Additive Explanations
TRIPOD: Transparent Reporting of a Multivariable Prediction Model for Individual Prognosis or Diagnosis
TSH: thyroid-stimulating hormone
UMP: follicular tumor of uncertain malignant potential

## References

[1] Boucai L, Zafereo M, Cabanillas ME (2024). Thyroid cancer: a review. JAMA.

[2] Grani G, Lamartina L, Durante C, Filetti S, Cooper DS (2018). Follicular thyroid cancer and Hürthle cell carcinoma: challenges in diagnosis, treatment, and clinical management. Lancet Diabetes Endocrinol.

[3] McHenry CR, Phitayakorn R (2011). Follicular adenoma and carcinoma of the thyroid gland. Oncologist.

[4] Haugen BR, Alexander EK, Bible KC (2016). 2015 American Thyroid Association management guidelines for adult patients with thyroid nodules and differentiated thyroid cancer: the American Thyroid Association Guidelines Task Force on thyroid nodules and differentiated thyroid cancer. Thyroid.

[5] Durante C, Hegedüs L, Czarniecka A (2023). 2023 European Thyroid Association Clinical Practice Guidelines for thyroid nodule management. Eur Thyroid J.

[6] Zachariah FJ, Rossi LA, Roberts LM, Bosserman LD (2022). Prospective comparison of medical oncologists and a machine learning model to predict 3-month mortality in patients with metastatic solid tumors. JAMA Netw Open.

[7] Farrokhian N, Holcomb AJ, Dimon E (2022). Development and validation of machine learning models for predicting occult nodal metastasis in early-stage oral cavity squamous cell carcinoma. JAMA Netw Open.

[8] Tseng YJ, Wang HY, Lin TW, Lu JJ, Hsieh CH, Liao CT (2020). Development of a machine learning model for survival risk stratification of patients with advanced oral cancer. JAMA Netw Open.

[9] Bennett M, Hayes K, Kleczyk EJ, Mehta R (2022). Similarities and differences between machine learning and traditional advanced statistical modeling in healthcare analytics. arXiv.

[10] Rajula HSR, Verlato G, Manchia M, Antonucci N, Fanos V (2020). Comparison of conventional statistical methods with machine learning in medicine: diagnosis, drug development, and treatment. Medicina (Kaunas).

[11] Bzdok D, Krzywinski M, Altman N (2017). Points of significance: machine learning: a primer. Nat Methods.

[12] Carmichael I, Marron JS (2018). Data science vs. statistics: two cultures?. Jpn J Stat Data Sci.

[13] Shin I, Kim YJ, Han K (2020). Application of machine learning to ultrasound images to differentiate follicular neoplasms of the thyroid gland. Ultrasonography.

[14] Tang P, Ren C, Shen L, Zhou Z (2021). Development and validation of a diagnostic nomogram for the preoperative differentiation between follicular thyroid carcinoma and follicular thyroid adenomas. J Comput Assist Tomogr.

[15] Lin AC, Liu Z, Lee J (2024). Generating a multimodal artificial intelligence model to differentiate benign and malignant follicular neoplasms of the thyroid: a proof-of-concept study. Surgery.

[16] Seo JK, Kim YJ, Kim KG, Shin I, Shin JH, Kwak JY (2017). Differentiation of the follicular neoplasm on the gray-scale US by image selection subsampling along with the marginal outline using convolutional neural network. Biomed Res Int.

[17] Yang Z, Yao S, Heng Y (2023). Automated diagnosis and management of follicular thyroid nodules based on the devised small-dataset interpretable foreground optimization network deep learning: a multicenter diagnostic study. Int J Surg.

[18] Li J, Li C, Zhou X (2023). US risk stratification system for follicular thyroid neoplasms. Radiology.

[19] Yang B, Yan M, Yan Z, Zhu C, Xu D, Dong F (2020). Segmentation and classification of thyroid follicular neoplasm using cascaded convolutional neural network. Phys Med Biol.

[20] Yang C, Kors JA, Ioannou S (2022). Trends in the conduct and reporting of clinical prediction model development and validation: a systematic review. J Am Med Inform Assoc.

[21] Paparodis R, Imam S, Todorova-Koteva K, Staii A, Jaume JC (2014). Hashimoto’s thyroiditis pathology and risk for thyroid cancer. Thyroid.

[22] MDT team of thyroid and parathyroid tumor [Article in Chinese]. Peking University Third Hospital.

[23] Collins GS, Reitsma JB, Altman DG, Moons KGM (2015). Transparent reporting of a multivariable prediction model for individual prognosis or diagnosis (TRIPOD): the TRIPOD statement. BMJ.

[24] Baloch ZW, Asa SL, Barletta JA (2022). Overview of the 2022 WHO classification of thyroid neoplasms. Endocr Pathol.

[25] Li X, Xiao WC, Mei F (2023). The association of pregnancy with disease progression in patients previously treated for differentiated thyroid cancer: a propensity score-matched retrospective cohort study. J Womens Health (Larchmt).

[26] Xiao WC, Li X, Shan R (2024). Pregnancy and progression of differentiated thyroid cancer: a propensity score-matched retrospective cohort study. J Clin Endocrinol Metab.

[27] Shan R, Li X, Tao M (2024). Pregnancy and the disease recurrence of patients previously treated for differentiated thyroid cancer: a systematic review and meta analysis. Chin Med J (Engl).

[28] Mincer DL, StatPearls J (2024). Treasure Island (FL) Ineligible Companies Disclosure: Ishwarlal Jialal Declares No Relevant Financial Relationships with Ineligible Companies.

[29] Ito Y, Miyauchi A, Fujishima M (2023). Thyroid-stimulating hormone, age, and tumor size are risk factors for progression during active surveillance of low-risk papillary thyroid microcarcinoma in adults. World J Surg.

[30] Taquet M, Griffiths K, Palmer EOC (2023). Early trajectory of clinical global impression as a transdiagnostic predictor of psychiatric hospitalisation: a retrospective cohort study. Lancet Psychiatry.

[31] Li X, Fu P, Xiao WC (2024). Associations of gestational thyrotropin levels with disease progression among pregnant women with differentiated thyroid cancer: a retrospective cohort study. Front Endocrinol (Lausanne).

[32] Kotthoff L, Sonabend R, Foss N, Bischl B, Bischl B, Sonabend R, Kotthoff L, Lang M (2024). Applied Machine Learning Using mlr3 in R.

[33] Pedregosa F, Varoquaux G, Gramfort A (2011). Scikit-learn: machine learning in Python. J Mach Learn Res.

[34] Lundberg SM, Erion G, Chen H (2020). From local explanations to global understanding with explainable AI for trees. Nat Mach Intell.

[35] Mukhopadhyay S (2022). InfoGram and admissible machine learning. Mach Learn.

[36] Mohammed R, Rawashdeh J, Abdullah M Machine learning with oversampling and undersampling techniques: overview study and experimental results.

[37] Saito T, Rehmsmeier M (2015). The precision-recall plot is more informative than the ROC plot when evaluating binary classifiers on imbalanced datasets. PLoS One.

[38] Liberati A, Altman DG, Tetzlaff J (2009). The PRISMA statement for reporting systematic reviews and meta-analyses of studies that evaluate healthcare interventions: explanation and elaboration. BMJ.

[39] Yang C, Fridgeirsson EA, Kors JA, Reps JM, Rijnbeek PR (2024). Impact of random oversampling and random undersampling on the performance of prediction models developed using observational health data. J Big Data.

[40] Elreedy D, Atiya AF, Kamalov F (2024). A theoretical distribution analysis of synthetic minority oversampling technique (SMOTE) for imbalanced learning. Mach Learn.

[41] Liu XY, Wu J, Zhou ZH (2009). Exploratory undersampling for class-imbalance learning. IEEE Trans Syst Man Cybern B Cybern.

[42] Riley RD, Archer L, Snell KIE (2024). Evaluation of clinical prediction models (part 2): how to undertake an external validation study. BMJ.

[43] Hashimoto DA, Varas J, Schwartz TA (2024). Practical guide to machine learning and artificial intelligence in surgical education research. JAMA Surg.

