# Supplementary material for: Interpretable Machine Learning to Predict the Malignancy Risk of Follicular Thyroid Neoplasms in Extremely Unbalanced Data: Retrospective Cohort Study and Literature Review
Source: JMIR Cancer. 2025 Feb 10;11:e66269. doi: 10.2196/66269 (PMC11833187; doi:10.2196/66269)
Supplement: Multimedia Appendix 1 [file cancer-v11-e66269-s001.docx]

1. **SHAP**

1-1. SHAP Summary Plot

SHAP Summary Plot shows the feature importance and effects of a dataset, using SHAP values (impact on model output). The x-axis represents the SHAP value, while each row on the y-axis represents a different feature. Each point in the plot corresponds to a SHAP value for a feature for an individual prediction. The position on the x-axis indicates the magnitude and direction of the feature's impact (positive or negative).

1-2. SHAP Interaction Value Dependence Plot (SHAP interaction plot)

A dependence plot visualizes the relationship of a single feature versus its SHAP interaction value. It reveals interaction effects with another feature. The x-axis represents the feature value, and the y-axis represents the SHAP interaction value. Points are colored by the value of another feature.

1. **Definitions of TSH related features**
2. Mean TSH score: interval-adjusted detailed TSH score[1]
3. tRMSSD of TSH: the time-adjusted root mean square of successive differences[2]
4. Mean TSH: mean value of preoperative TSH
5. Standard deviation of TSH: standard deviation of preoperative TSH
6. Coefficient of variation of TSH: Standard deviation of TSH/ Mean TSH
7. **Figure S1: F-TIRADS scoring criteria[3]**


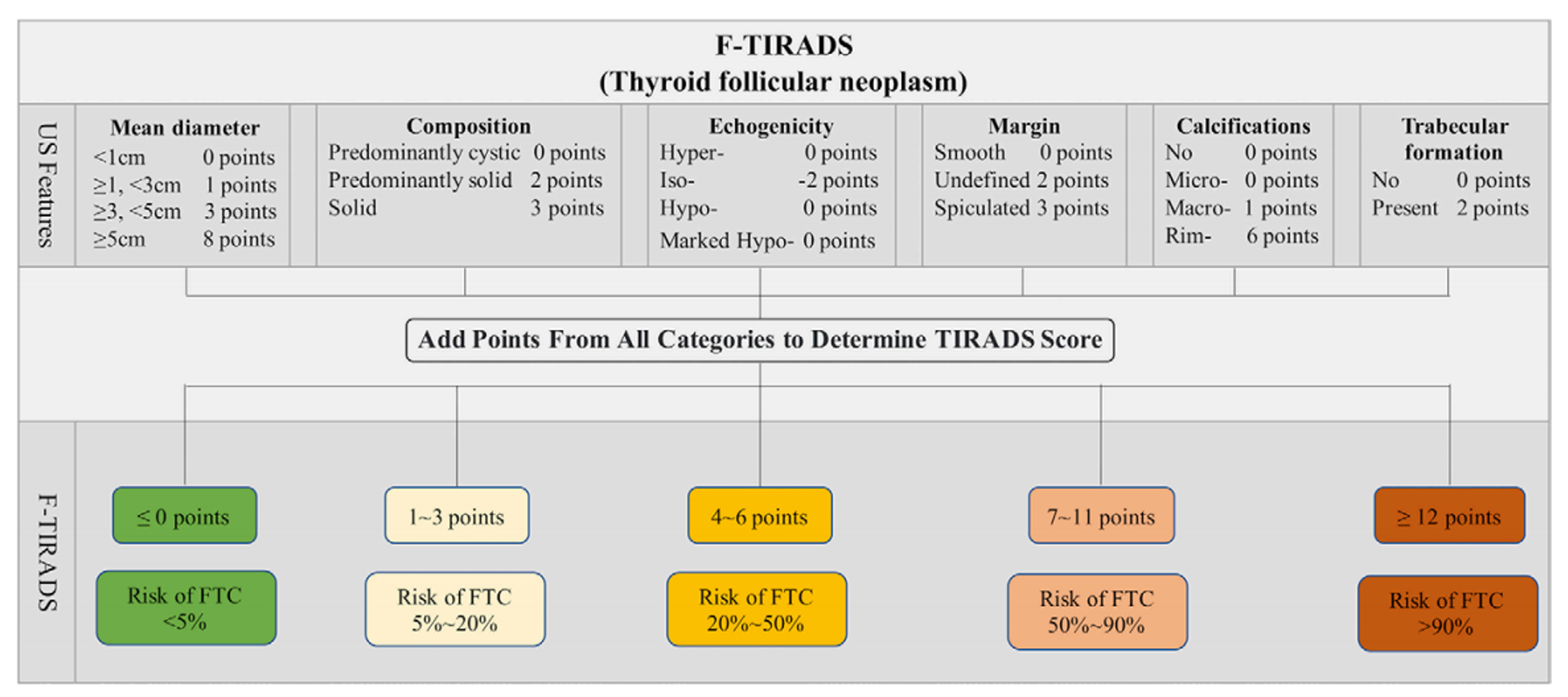


Figure S1: Chart shows the six categories of follicular neoplasms associated with the US follicular Thyroid Imaging Reporting and Data System (F-TIRADS), with fitted probabilities and indications for follicular thyroid carcinoma (FTC).

**Reference:**

1. Ito Y, Miyauchi A, Fujishima M, Noda T, Sano T, Sasaki T, et al. Thyroid-Stimulating Hormone, Age, and Tumor Size are Risk Factors for Progression During Active Surveillance of Low-Risk Papillary Thyroid Microcarcinoma in Adults. World J Surg. 2023 Feb;47(2):392-401. PMID: 36182976. doi: 10.1007/s00268-022-06770-z.

2. Taquet M, Griffiths K, Palmer EOC, Ker S, Liman C, Wee SN, et al. Early trajectory of clinical global impression as a transdiagnostic predictor of psychiatric hospitalisation: a retrospective cohort study. Lancet Psychiatry. 2023 May;10(5):334-41. PMID: 36966787. doi: 10.1016/s2215-0366(23)00066-4.

3. Li J, Li C, Zhou X, Huang J, Yang P, Cang Y, et al. US Risk Stratification System for Follicular Thyroid Neoplasms. Radiology. 2023 Nov;309(2):e230949. PMID: 37987664. doi: 10.1148/radiol.230949.
