# Supplementary material for: Interpretable Machine Learning to Predict the Malignancy Risk of Follicular Thyroid Neoplasms in Extremely Unbalanced Data: Retrospective Cohort Study and Literature Review
Source: JMIR Cancer. 2025 Feb 10;11:e66269. doi: 10.2196/66269 (PMC11833187; doi:10.2196/66269)
Supplement: Multimedia Appendix 2 [file cancer-v11-e66269-s002.docx]

**Search Strategy**

1. **PubMed**

(("adenocarcinoma, follicular"[MeSH Terms] OR ("adenocarcinoma"[All Fields] AND "follicular"[All Fields]) OR "follicular adenocarcinoma"[All Fields] OR ("follicular"[All Fields] AND "thyroid"[All Fields] AND "cancer"[All Fields]) OR "follicular thyroid cancer"[All Fields]) AND "predict*"[All Fields])

1. **Web of Science**

((TS=(follicular thyroid cancer)) OR TS=(follicular thyroid carcinoma)) AND TS=(predict*)

1. **Embase**

#3. #1 AND #2

#2. 'prediction'

#1. 'thyroid follicular carcinoma'/exp OR 'thyroid

follicular carcinoma'

1. **IEEE Xplore**

("All Metadata":thyroid cancer) AND ("All Metadata":predict)

**Eligibility Criteria**

**Inclusion Criteria:**

1. Studies focused on preoperative prediction of the benign or malignant nature of follicular thyroid neoplasms (FTNs).
2. The proportion of patients with FTNs in the study population must exceed 50%.
3. The study utilized traditional statistical models, machine learning, deep learning, etc. to develop predictive models.

**Exclusion Criteria:**

1. Reviews or study protocols.
2. Studies published in languages other than English.
