# Supplementary figures and images for: Interpretable Machine Learning to Predict the Malignancy Risk of Follicular Thyroid Neoplasms in Extremely Unbalanced Data: Retrospective Cohort Study and Literature Review
Source: JMIR Cancer. 2025 Feb 10;11:e66269. doi: 10.2196/66269 (PMC11833187; doi:10.2196/66269)

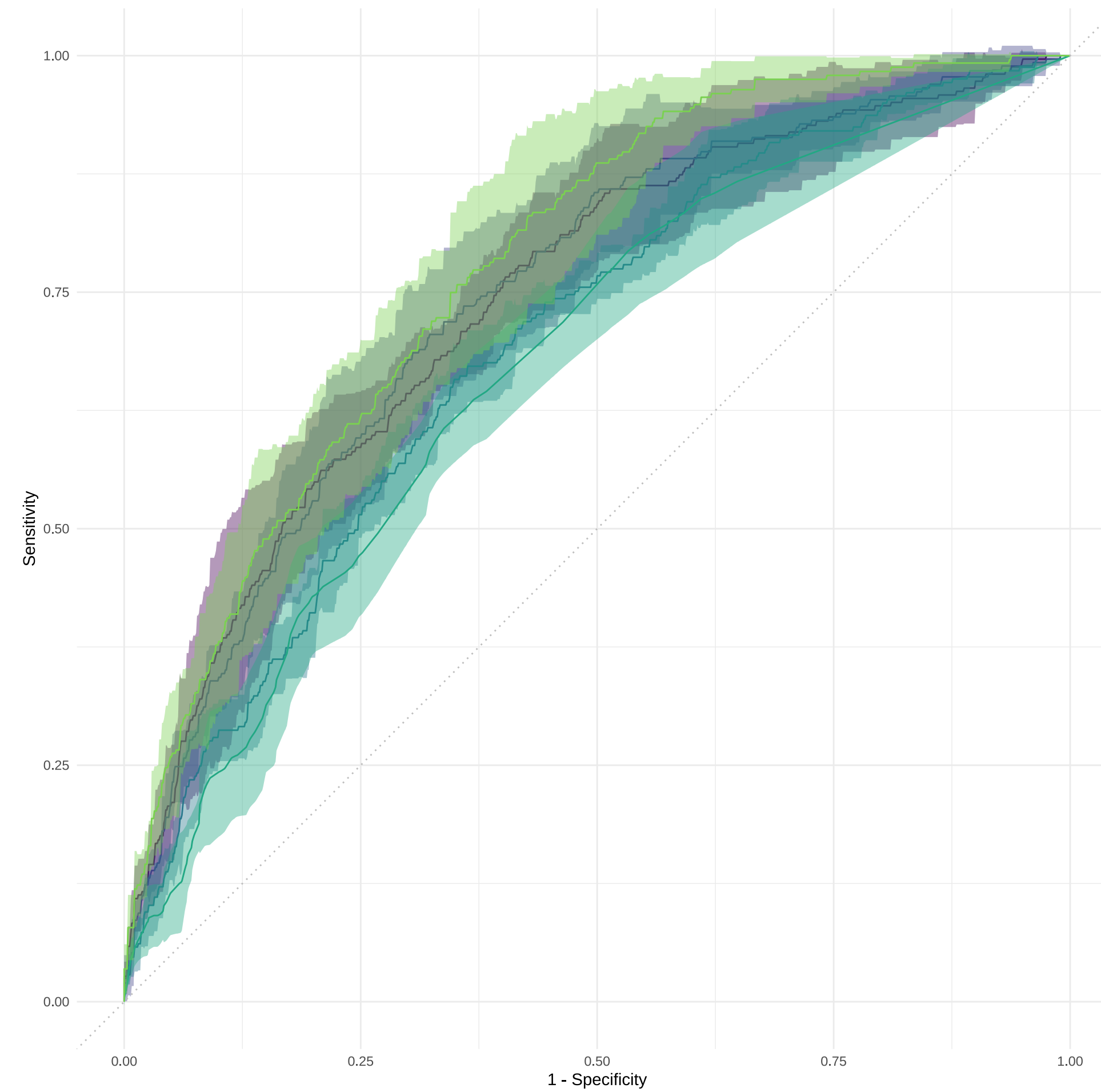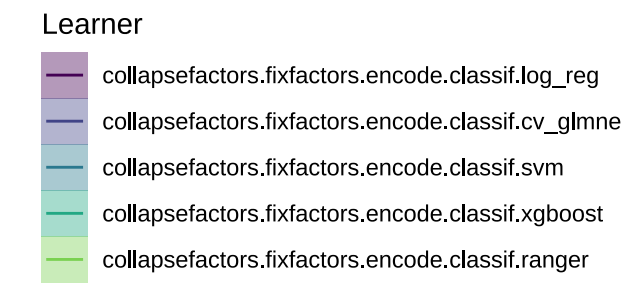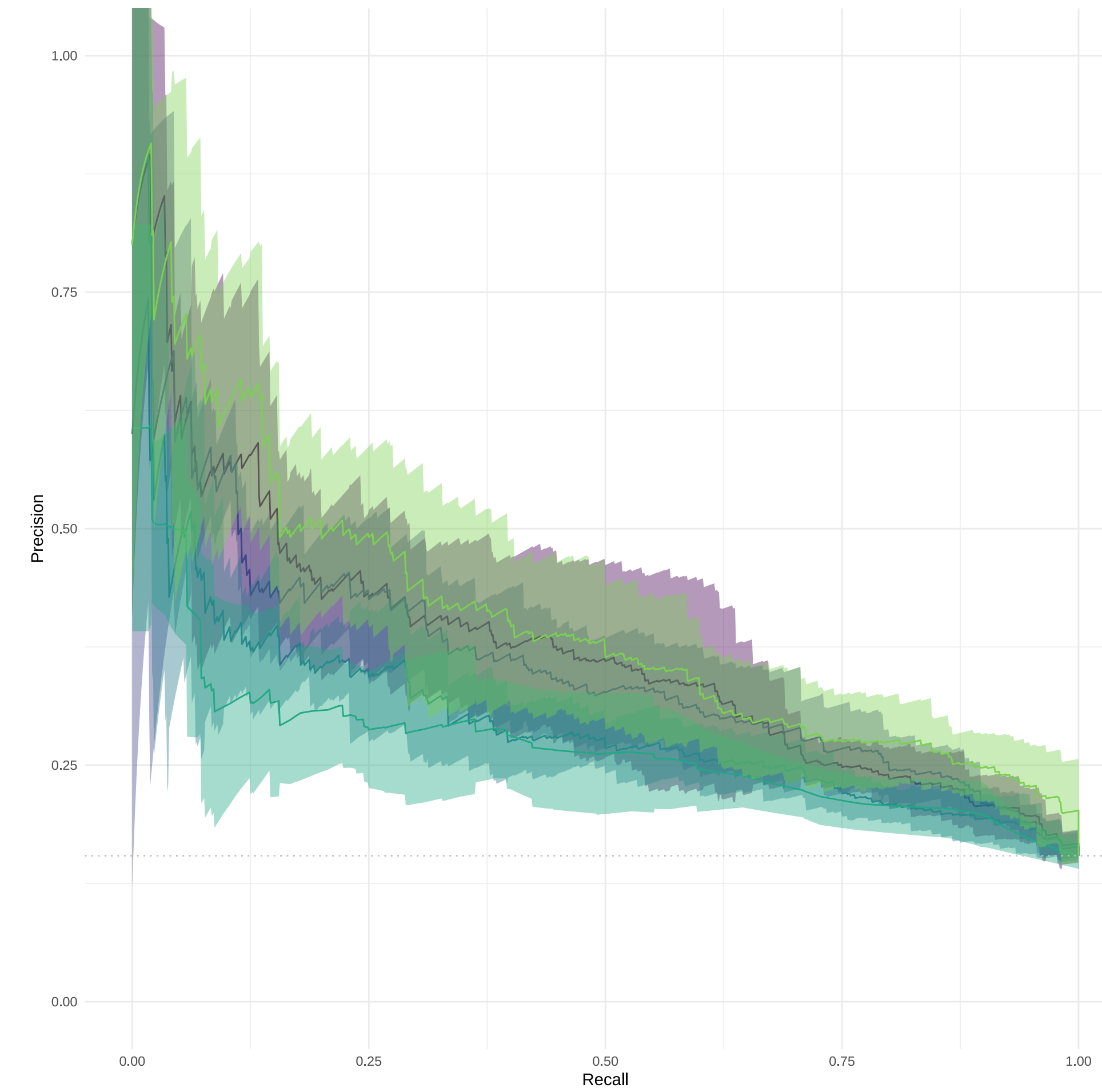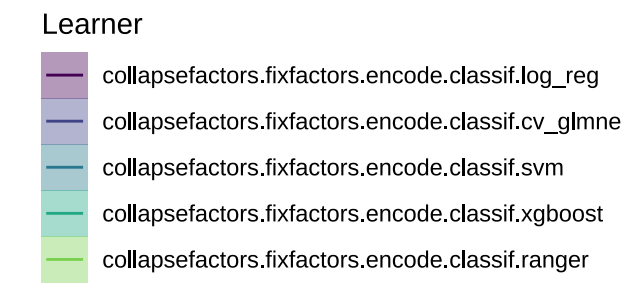

Supplement: Multimedia Appendix 3 [file cancer-v11-e66269-s003.pdf]

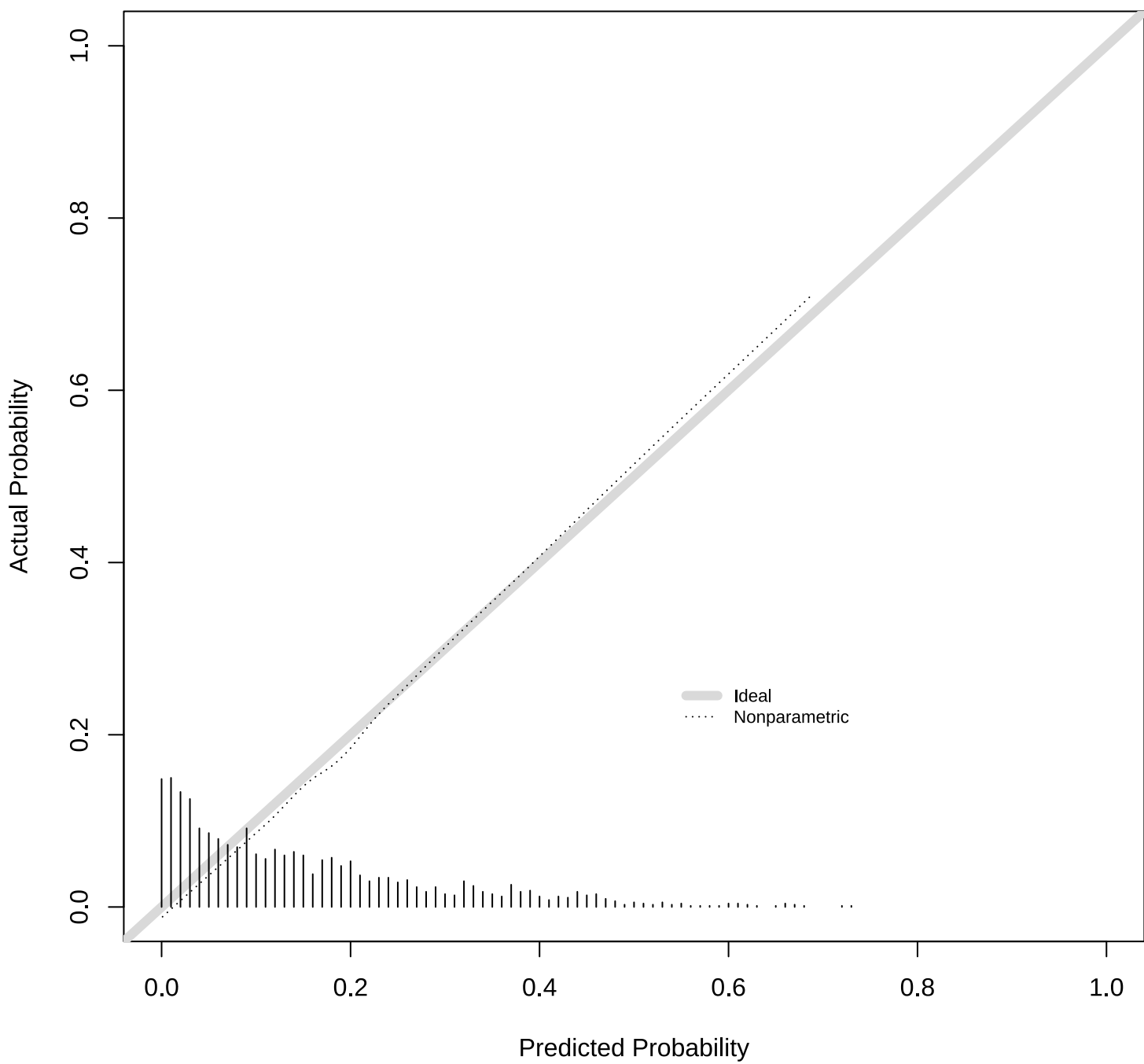

Supplement: Multimedia Appendix 6 [file cancer-v11-e66269-s006.pdf]

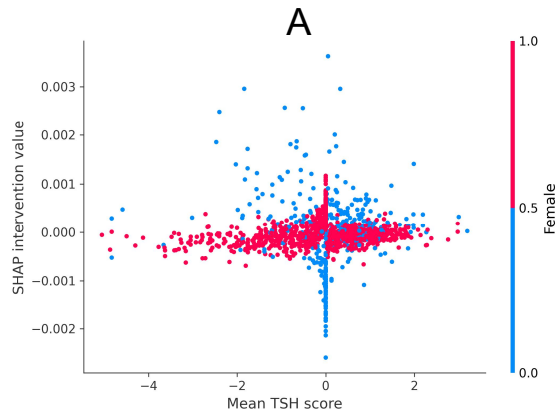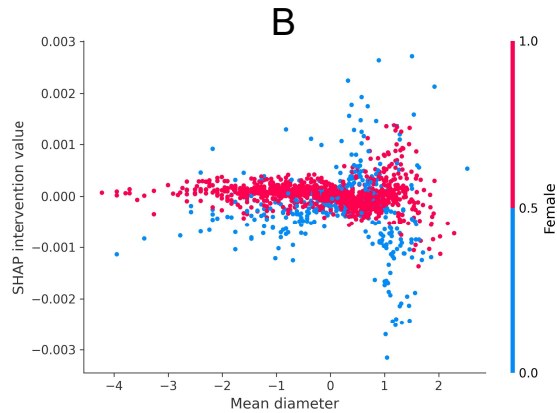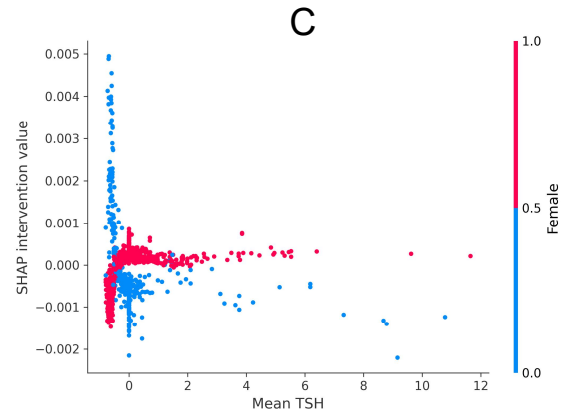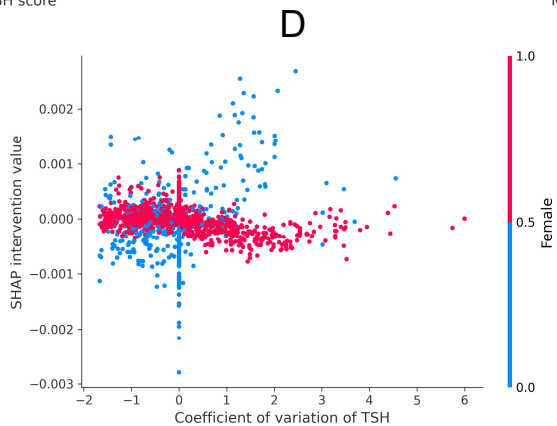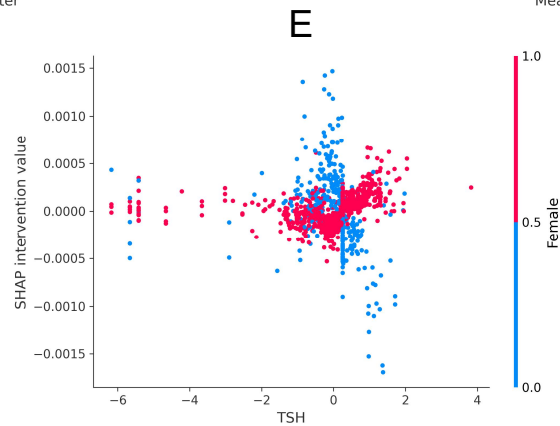

Supplement: Multimedia Appendix 7 [file cancer-v11-e66269-s007.pdf]

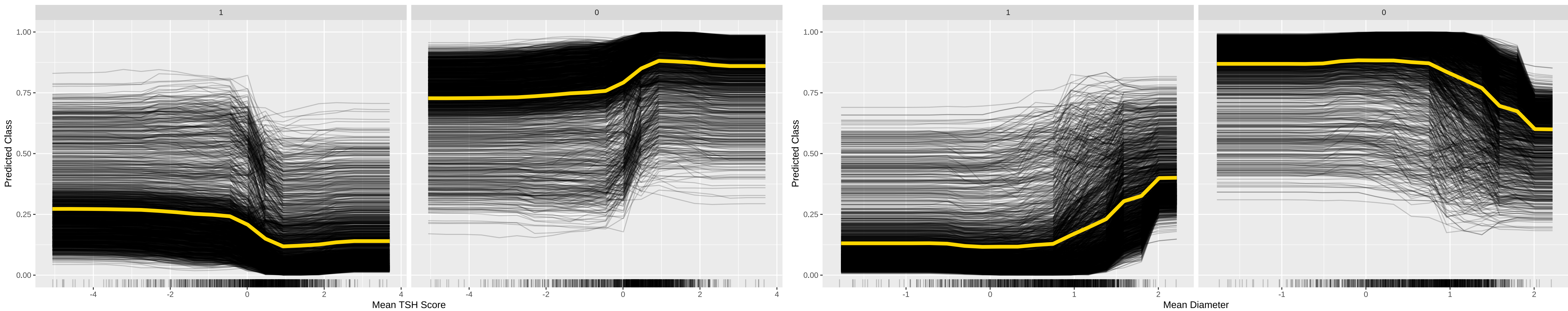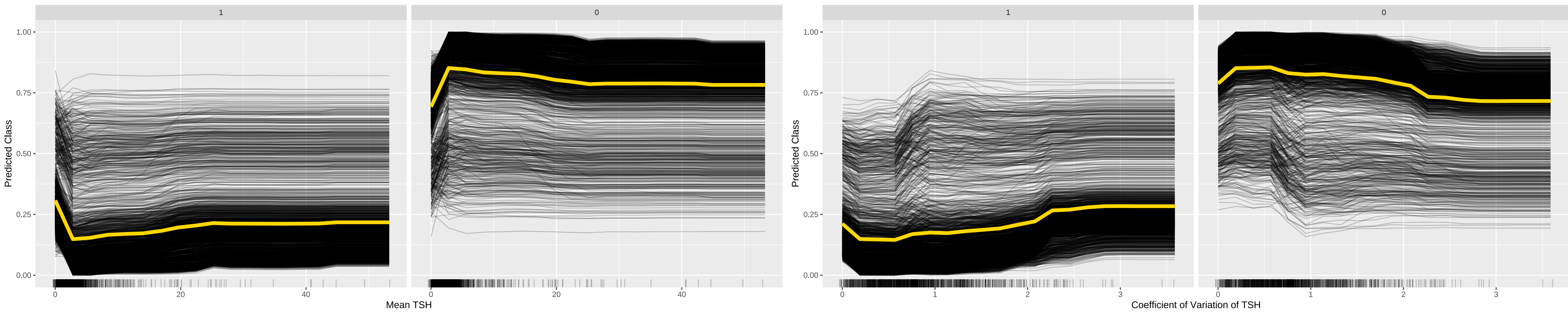

Supplement: Multimedia Appendix 8 [file cancer-v11-e66269-s008.pdf]
