# Supplementary material for: Interpretable Machine Learning to Predict the Malignancy Risk of Follicular Thyroid Neoplasms in Extremely Unbalanced Data: Retrospective Cohort Study and Literature Review
Source: JMIR Cancer. 2025 Feb 10;11:e66269. doi: 10.2196/66269 (PMC11833187; doi:10.2196/66269)
Supplement: Multimedia Appendix 4 [file cancer-v11-e66269-s004.docx]

| Model | AUROC^a^ | AUPRC^b^ | Accuracy | Sensitivity | Precision | Specificity |
| --- | --- | --- | --- | --- | --- | --- |
| Random forest | 0.79 | 0.40 | 0.85 | 0.07 | 0.58 | 0.99 |
| Lasso regression | 0.73 | 0.35 | 0.85 | 0.02 | NA^c^ | 0.998 |
| Logistic regression | 0.74 | 0.38 | 0.85 | 0.15 | 0.50 | 0.97 |
| Support Vector Machine | 0.71 | 0.33 | 0.85 | 0.04 | 0.55 | 0.99 |
| XGBoost | 0.69 | 0.29 | 0.82 | 0.18 | 0.35 | 0.94 |

^a^AUROC= Area under the receiver operator characteristic curve; ^b^AUPRC= Area under the precision-recall curve**;** ^c^NA=data not available.

**Supplemental Table2. AUC and Confusion Matrix (True positive, True negative, False positive, False negative).**

| Threshold-free Parameter | Value | | 95% CI^a^ | |
| --- | --- | --- | --- | --- |
| AUROC^b^ | 0.79 | | 0.77-0.81 | |
| AUPRC^c^ | 0.40 | | 0.37-0.44 | |
| Confusion Matrix 1: Threshold 50% | | | | |
|  | Truth | | | |
| Prediction |  | FTA^d^ | | FTC^e^ |
|  | FTA | 1401 | | 240 |
|  | FTC | 13 | | 18 |
| Parameters | Accuracy | 0.85 | |  |
|  | Sensitivity | 0.07 | |  |
|  | Specificity | 0.99 | |  |
|  | Precision | 0.58 | |  |
| Confusion Matrix 2: Threshold 40% | | | | |
|  | Truth | | | |
| Prediction |  | FTA | | FTC |
|  | FTA | 1361 | | 205 |
|  | FTC | 53 | | 53 |
| Parameters | Accuracy | 0.85 | |  |
|  | Sensitivity | 0.21 | |  |
|  | Specificity | 0.96 | |  |
|  | Precision | 0.50 | |  |
| Confusion Matrix 3: Threshold 30% | | | | |
|  | Truth | | | |
| Prediction |  | FTA | | FTC |
|  | FTA | 1261 | | 147 |
|  | FTC | 153 | | 111 |
| Parameters | Accuracy | 0.82 | |  |
|  | Sensitivity | 0.43 | |  |
|  | Specificity | 0.89 | |  |
|  | Precision | 0.42 | |  |
| Confusion Matrix 4: Threshold 20% | | | | |
|  |  | Truth | |  |
| Prediction |  | FTA | | FTC |
|  | FTA | 1048 | | 82 |
|  | FTC | 366 | | 176 |
| Parameters | Accuracy | 0.73 | |  |
|  | Sensitivity | 0.68 | |  |
|  | Specificity | 0.74 | |  |
|  | Precision | 0.32 | |  |
| Confusion Matrix 5: Threshold 10% | | | | |
|  |  | Truth | |  |
| Prediction |  | FTA | | FTC |
|  | FTA | 636 | | 22 |
|  | FTC | 778 | | 236 |
| Parameters | Accuracy | 0.52 | |  |
|  | Sensitivity | 0.91 | |  |
|  | Specificity | 0.45 | |  |
|  | Precision | 0.23 | |  |

^a^95% CI: 95% confidence interval; ^b^AUROC: Area under the receiver operator characteristic curve; ^c^AUPRC: Area under the precision-recall curve; ^d^FTA: Follicular thyroid adenoma; ^e^FTC: Follicular thyroid carcinoma.
